# “Digital biomarkers” in preclinical heart failure models — a further step towards improved translational research

**DOI:** 10.1007/s10741-022-10264-4

**Published:** 2022-08-24

**Authors:** Alexander Schmidt, Jakob Balitzki, Ljubica Grmaca, Julia Vogel, Philip Boehme, Katharina Boden, Jörg Hüser, Hubert Truebel, Thomas Mondritzki

**Affiliations:** 1 Bayer AG, BAG-PH-RD-RED-TA1-CPM-CPM2, Building 0520, 42096 Wuppertal, Germany; 2 Heinrich-Heine-University, Düsseldorf, Germany; 3 Hannover Medical School, Hannover, Germany; 4 Philipps-University of Marburg, Marburg, Germany; 5 University of Witten/Herdecke, Witten, Germany; 6 Clinic for Cardiology and Angiology, West-German Heart and Vascular Center, Faculty of Medicine, University Duisburg-Essen, Duisburg, Germany

**Keywords:** Heart failure, Animal model, Mobile health, Telemetry, Translational research

## Abstract

Innovations in the development of novel heart failure therapies are essential to further increase the predictive value of early research findings. Animal models are still playing a pivotal role in ‘translational research’. In recent years, the transferability from animal studies has been more and more critically discussed due to persistent high attrition rates in clinical trials. However, there is an increasing trend to implement mobile health devices in preclinical studies. These devices can increase the predictive value of animal models by providing more accurate and translatable data and protect from confounding factors. This review outlines the current prevalence and opportunities of these techniques in preclinical heart failure research studies to accelerate the integration of these important tools. A literature screening for preclinical heart failure studies in large animals implementing telemetry devices over the last decade was performed. Twelve out of 43 publications were included. A variety of different hemodynamic and cardiac parameters can be recorded in conscious state by means of telemetry devices in both, the animal model and the patient. The measurement quality is consistently rated as valid and robust. Mobile health technologies functioning as digital biomarkers represent a more predictive approach compared to the traditionally used invasive measurement techniques, due to the possibility of continuous data collection in the conscious animal. Furthermore, they help to implement the 3R concept (reduction, refinement, replacement) in animal research. Despite this, the use of these techniques in preclinical research has been restrained to date.

## Introduction

Heart failure (HF) has become a global pandemic with an increasing prevalence, an estimated 26 million people suffering worldwide [1, 2]. It is expected that the overall prevalence of HF will increase 2.3-fold by the year 2040 and will triple by the year 2060 [3]. Mortality is high as 50% of patients die within 5 years and 90% within 10 years after diagnosis [4]. Numerous hospitalizations are also a reason for the severely reduced quality of life of HF patients [5]. Despite this still high unmet medical need, the willingness of the pharmaceutical industry to invest in new therapies for cardiovascular (CV) indications has stagnated [6]. There are several possible reasons for this: Basically, the development of new therapies for CV diseases is more expensive than for other indications [7]. Late-stage drug development costs are a major contributor to the overall costs in research and development (R&D) as clinical studies must show an additional benefit compared to standard therapy measured by hard endpoints, like mortality or hospitalization. This requires very cost-intensive long-term studies including large numbers of multimorbid patients that are necessary to reach these endpoints [7]. To minimize failures during this critical phase of the drug development process, innovative approaches are of great importance. One such approach is to improve the predictivity of preclinical research by incorporating translational research. Translational research is defined as the integration of early basic and clinical research and has the primary goal to reach clinical endpoints of pivotal studies with greater certainty [8]. Thus, further investments are saved, and available funds can be used in the best possible way. In addition, translational research contributes to risk reduction for patients or healthy volunteers during subsequent clinical trials [9].

The majority of early basic research during drug development is based on animal (disease) models. In 2009, a translatability score was designed by Wehling to assess the risk and potential of a given pharmaceutical development project [10]. In this score, animal models are highlighted as an essential component of the drug discovery process depending on their physiological relevance and prediction of the human disease. The better an animal model is characterized, the higher the chance that the results will be transferred into the late phase of drug development and into clinical practice.

Especially large animal (LA) models are of great importance for drug development but also for the development of new medical devices and surgical procedures. While small animal (SA) models are indispensable tools to perform proof-of-concept studies quickly and relatively inexpensively with a certain degree of statistical confidence, they show significant limitations and should therefore be interpreted with caution [11]. Besides the limitations in emulating the patient pathophysiology, SAs also have profound physiological differences compared to humans. Rodent contraction kinetics for example, result in a significantly higher heart rate (HR) compared to humans (60–80 bpm in men vs. 500–800 bpm in mice), limiting their cardiac reserve [11, 12]. The homogeneous genetic background of rodents also contrasts with the heterogeneity of humans and the resulting phenotyping of diseases [11].

On the opposite, it is well known that LA models for example for aortic stenosis or mitral regurgitation more realistically describe critical structural and functional aspects of the clinical phenotype and thus the development of HF resulting in an increased translational value [13]. Therefore, results from SA models should be verified in LA models before a new therapy reaches a clinical trial. Thus, LA models are of great importance for the validation of new therapy options and will be the focus of this review.

There are many examples where the translation from basic research into clinical trials has failed [14]. Obviously, factors such as study design, experimental execution and data interpretation influence the translational value. Today, invasive diagnostic methods and the necessity of examination under resting conditions typically requires anesthesia during animal experiments [15]. However, as early as 1975, Vatner and Braunwald were able to show that “*the assumption that anesthesia and surgical trauma exert only minor effects on the response to physiologic and pharmacologic interventions is not tenable*” [16]. Their findings in narcotized animals were substantially different from those obtained in experiments with conscious animals [16]. Thus, the authors have demonstrated that general anesthesia is a confounder for hemodynamic and cardiac investigations, then called “*physiological reactance*” [16]. In recent years, new digital technologies, such as radio-telemetry devices, have been developed which could be helpful to protect from this confounder and thereby increase the translational value of animal studies.

**Fig. 1:**
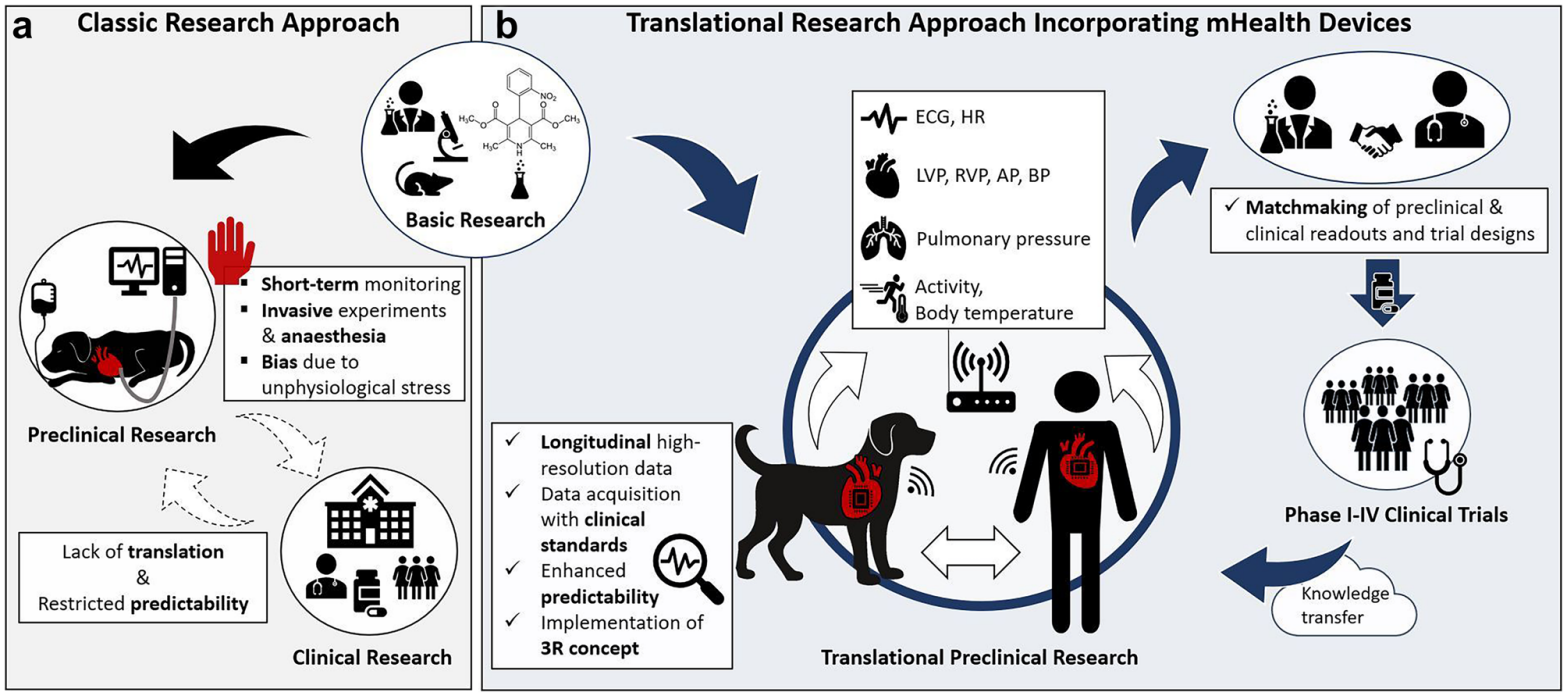
mHealth devices in the rationale of translational research. **a** Classical path of drug development and its limitations. **b** Integration of mHealth devices in large animal disease models and patients — possibilities and postulated benefits. 3R, Reduction, Replacement, Refinement; AP, Aortic pressure; BP, Blood pressure; ECG, Electrocardiogram; HR, Heart rate; LVP, Left ventricular pressure; mHealth, Mobile health; RVP, Right ventricular pressure

Digital technologies are on the rise and could also revolutionize the healthcare sector [17]. Besides genome analysis and artificial intelligence approaches, mobile health (mhealth) technologies are most prominently discussed approaches for the future medical environment. The term “mhealth” describes portable devices supporting the medical practice [18]. Different types of mhealth technologies can be found: smartphone apps and smartphone-connected devices, wearable and wireless devices, handheld-imaging platforms and sensor-based technologies [18]. Biomarkers could help to identify patients having the respective disease or they could help to detect early signs of efficacy or safety in clinical trials [19]. Besides molecular biomarkers, new approaches using digital biosensors, wearable or implantable devices are used to collect functional readouts in clinical trials and practice [20–22]. Some of these approaches may have the benefit to improve adherence to medication and quality of care, decrease hospitalization and medical visits and ultimately decrease health care costs [23–27]. For instance, pulmonary pressure-guided HF management, using a remote monitoring approach with a small implantable sensor leads to a decrease in morbidity and mortality in patients suffering from HF with reduced ejection fraction [28]. But mhealth devices can not only support clinical practice, but also improve the translational value of preclinical research into outcomes of clinical trials and thus increase the predictive value for efficacy and safety of new therapies for different reasons (Fig. 1): They may provide more human disease relevant readouts, they allow the collection of diagnostic, prognostic and safety readouts in larger quantity and higher quality and they enable long-term and continuous data recording [29, 30]. In addition, in animal studies, mhealth devices help to implement the 3R (reduction, refinement, replacement) concept, i.e., to further reduce, replace and refine the use of animals in research [31]. Different dosages or routes of administration can be consecutively performed in the same animal utilizing telemetry devices, thereby replacing conventional study designs. Besides, bias due to inter-animal variation is avoided by this “blocked study design” [31].

In the end, mhealth technologies could function as a “digital biomarker” to improve the translational value of animal models by continuous data recording in conscious animals and thus make late-stage development failures of new therapies in CV indications less likely. The aim of this review is to point out to which extent novel mhealth devices are used in preclinical HF research to eliminate the confounding factor “anesthesia”, which was described decades ago by Vatner and Braunwald [16]. For this purpose, a literature screening was performed. The results will enable to discuss and evaluate the aforementioned possibilities and benefits of digital biomarkers compared to standard methods using an anesthetic regime.

## Methods

In order to find relevant literature, the database Medline (PubMed) was searched for publications about LA studies in HF. Studies in this regard should be conducted in telemetered LAs. The following search equation (Eq. 1) was formulated by applying the advanced search builder: ‘(heart failure) AND (telemetry OR telemetric OR wearable electronic devices) AND (canine OR pig OR cat OR sheep OR goats OR large animals)’. The objective was to gain an overview of the use of novel mhealth devices. Therefore, the publication period was limited to the last 10 years. From received publications, those fitting to any of the following criteria were excluded: SA studies; clinical trials not including animal experiments; absence of a HF model; absence of wireless data transfer of hemodynamic or cardiac parameters. In addition Eq. 1 was changed to ‘((heart failure) AND (canine OR pig OR cat OR sheep OR goats OR large animals)) NOT (telemetry OR telemetric OR wearable electronic devices)’ to estimate the number of publications not utilizing telemetry devices from 2010 to 2020. The number of results obtained applying both search equations was plotted over time using Graphpad Prism Version 9.

After application of exclusion criteria, the remaining literature obtained with Eq. 1 was scanned according to predefined criteria. As basic information, the publication date and the first author were listed. The following aspects were then examined in terms of content: The species used, state of consciousness during data acquisition, disease status, interventions performed, whether the device was implanted or worn, the readouts recorded by the device and the aim of the study (efficacy, safety, methodical or other aims). Publication aims were classified as “efficacy” if the effect of a drug was being investigated. “Safety” referred to publications in the field of safety pharmacology investigations. Publications investigating disease models or the functionality and safety of mhealth devices were listed as “methodical”. Publications not corresponding to the aforementioned aims were summarized under “other aims”. In addition, model and manufacturer of the telemetry devices were included. The percentage distribution of study aims was plotted using Graphpad Prism Version 9.

## Results

### Results of literature screening

**Fig. 2:**
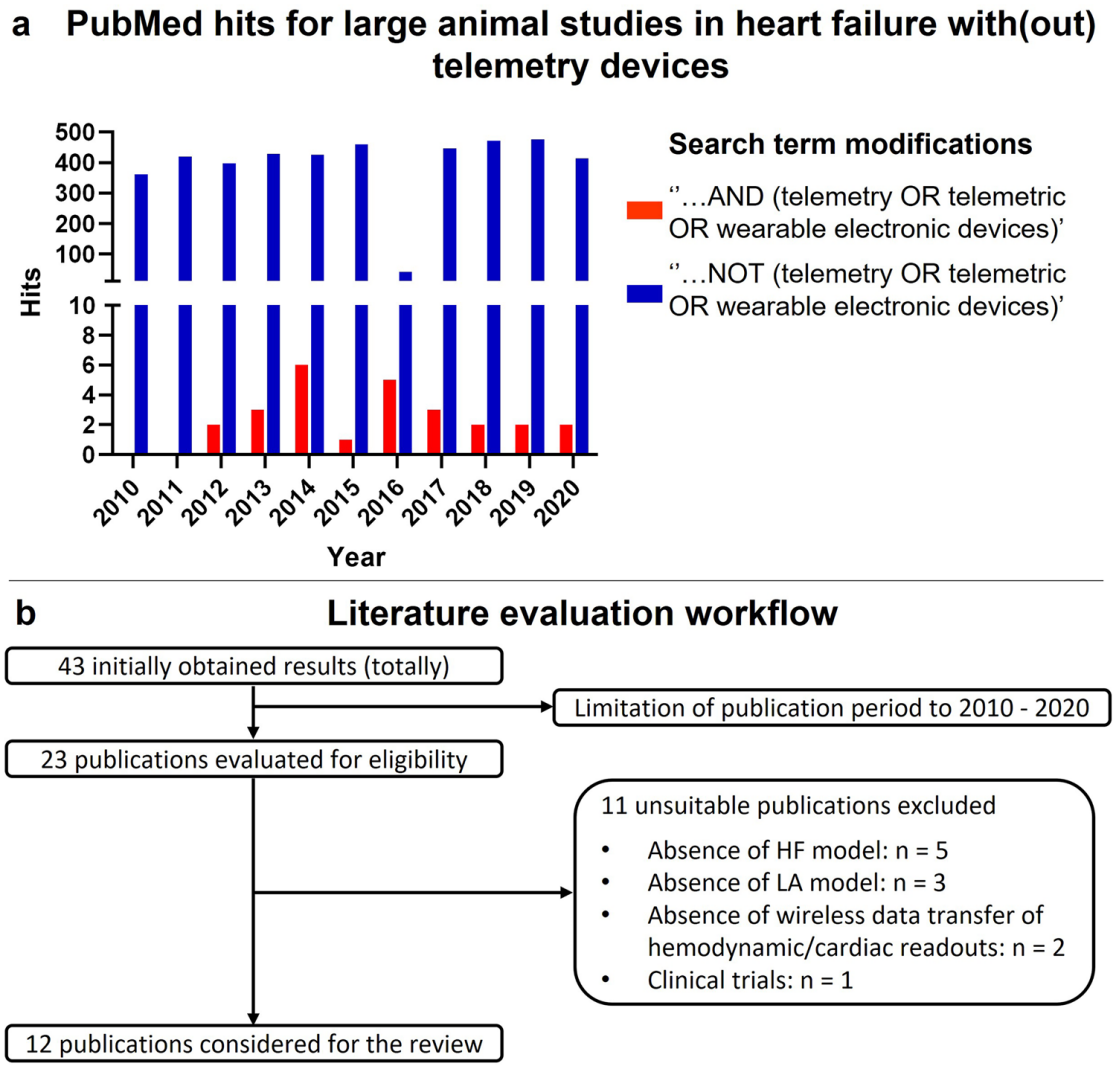
**a** Number of publications describing LA studies with (red bar) or without (blue bar) using telemetry devices. **b** Flowchart describing the workflow of literature evaluation applying search Eq. 1 combined with pre-defined exclusion criteria. Abbreviations: HF, Heart failure; LA, Large animal

The literature was screened in December 2020 according to the workflow shown in Fig. 2b. Application of the search equation resulted in 43 publications in total. After limiting the publication period to the years 2010–2020, 23 results remained for further analysis. By excluding publications that did not meet the inclusion criteria, 12 publications remained to be considered in the review. An overview of these publications is given in Table 1. Application of both search equations resulted in an overview of the number of publications (not) utilizing telemetry devices (Fig. 2a). This approach allows estimating that from 2010 to 2020, the vast majority of publications did not utilize telemetry devices (4102 vs. 23 hits).

**Table 1:** Overview of publications considered in this review including study design and aim as well as details of the telemetry device and the parameters assessed

**Publication**			**Readout(s)**	**Species**	**Awareness**	**Disease status**	**Intervention/compound tested**	**Aim**	**Device benefit**	**Device manu-facturer/model**	**Imp./wear**	**Short summary**
**First author(s)**	**Year**	**Ref.**										
Mondritzki T	2020	[32]	AP, CO (wear.), ECG, LVP (imp.)	*Beagle dog*	Con.	Healthy/ tachy-pacing-induced HF	Pecavaptan (BAY 1,753,011) vs. tolvaptan	Eff.	Delivers a more predictive model than the invasive measurement also performed	DSI/ L21, Cheetah medical/ NIKOM	Imp. + wear	Comparing the novel dual V1a/V2 receptor antagonist pecavaptan with the selective V2 receptor antagonist tolvaptan in preclinical HF models
Liu Z	2019	[33]	ECG	*Yorkshire pig*	Con.	Burst pacing-induced AF and HF	/	Other	Clinically relevant model combined with previously reported atrial gene painting technique	N.s	Imp.	Evaluating the effects of CaMKII on AF-related structural remodeling by transgene insert of human CaMKII
Perl L	2019	[34]	LAP	*Domestic sheep*	Anest.	Healthy	Phenylephrine	Meth.	Direct LAP measurement allows more accurate assessment of pulmonary congestion than PAP measurement	V-Lap^™^/ V-Lap	Imp.	Safety and feasibility test of a novel battery-less and wireless monitoring system to accurately measure LAP compared to direct invasive PCWP measurement
Mondritzki T	2018	[35]	Contractility, HR, LVEDP, LVSP, relaxation, TAU	*Beagle dog*	Con.	Pacing-induced HF	Dobutamine/vasopressin	Meth.	Direct LVP measurement using microelectromechanical sensors allowing earlier detection and more specific diagnosis of LV dysfunction than right heart or pulmonary measurements	LV-MEMS/ St. Jude Medical	Imp.	Preclinical study validating a new battery-less and easy to implant LV-microelectromechanical system to assess LV performance in a HF model
Malinowski M	2017	[36]	HR, LVEDP, LVESP, max. LVP, RVEDP, RVESP, max. RVP	*Dorset sheep*	Con.	HF with FTR (TIC) high-rate pacing-induced	LVAD	Meth.	Developed model serves as translational research platform to study the pathogenesis of FTR and elucidate the effects of a LVAD on RV function/mechanics	Transonic EndoGear Inc./ Endogear^®^ biotelemetry system	Imp.	Establishing a LA model of FTR, reflecting the clinical situation of end-stage HF patients for investigating it´s pathogenesis and elucidating the effect of mechanical LV support on RV function and mechanics
Regan C	2016	[37]	AP, DAP, ECG, LVP, LVWT	*Beagle dog*	Con.	Rapid ventricular pacing-induced HF	Pimobendan (Vetmedin^®^)	Meth.	PV-loop measurements without the confounder anesthesia in a model of cardiac dysfunction	Konigsberg Instruments/ ITS T27-G series	Imp.	Describes a methodology using a combination of telemetry and direct signal acquisition to record concomitant peripheral hemodynamics, ECG, and LV structure and function, including LV PV-loops to determine load independent measures of contractility in conscious dogs
Schwarzl M	2016	[38]	ECG	*Landrace pig*	Con.	AF by rapid atrial pacing/ HT by DOCA	/	Meth.	Pacemaker with simultaneous monitoring function of atrial rhythm in the awake animal to study the early phase of atrial remodeling	Customized	Imp.	Model of early atrial remodeling using a custom-built pacemaker allowing to characterize effects of maladaptive stimuli or protective interventions specifically during early AF
Choy J	2014	[15]	ECG, LAD/BC-flow, LVP, MAP	*Yorkshire pig*	Con.	HF by AV fistula, aortic banding, pacing-induced tachycardia	/	Meth.	First use of a multichannel system implanted in a LA HF model	Transonic EndoGear Inc./ EndoGear1	Imp.	Test of a telemetry system providing continuous recording of pressure, flow and ECG signals throughout the progression of HF in a swine model
Ewart L	2014	[39]	ECG (HR, QTc, PR), DBP	*Canine*	Con.	N.s	113 small molecule compounds	Saf.	Correlation of telemetrically collected data with results from phase I clinical studies	N.s	Imp.	Cross-company initiative to determine concordance between the conscious telemetered dog and phase I outcomes for 3 CV parameters
Caruso A	2014	[40]	BP (*monkey*: femoral artery; *dog*: aorta), ECG, LVP	*Beagle dog/Cynomolgus monkey*	Con.	Healthy	12 developmental compounds	Saf.	PK/PD modeling with telemetrically collected data	Different devices	Imp.	12 developmental compounds from diverse therapeutic areas were tested in CV safety studies in telemetered dogs and cynomolgus monkeys; 2 compounds were compared with clinical data
Asgari S	2014	[41]	HR, RPM, power (Watts)	*Swine*	Anest.	Healthy	LVAD	Meth.	Extension of an LVAD with telemetric monitoring and control capability. In addition, wireless power supply	N.s./ UMC-Physio	Imp.	Proof of concept study investigating feasibility and implantability of a LVAD with telemetric monitoring and regulation
Amir O	2013	[42]	Pulmonary fluid concentration	*Swine/human*	Anest.	Anterior myocardial infarction-induced HF	Overload isotonic saline and furosemide (subsequently)	Meth.	Evaluation of pulmonary congestion without the need for a CT	Sensible Medical Innovations Ltd/ ReDS technology	Wear	Test of a non-invasive electromagnetic monitoring system to detect changes in pulmonary fluid and pulmonary congestion

*AF* atrial fibrillation, *Anest.* Anesthetized, *AP* aortic pressure, *AV* atrioventricular, *BC-Flow* brachiocephalic flow, *BP* blood pressure, *BT* body temperature, *CaMKII* Ca2 + -/calmodulin-dependent protein kinase II, *CHF* chronic heart failure, *CT* computer tomography, *CO* cardiac output, *Con*. conscious, *CV* cardiovascular,* DAP* anterior–posterior left ventricular chamber diameter, *DBP* diastolic blood pressure, *DOCA* deoxycorticosterone acetate, *ECG* electrocardiogram, *Eff.* efficiency, *FTR* functional tricuspid regurgitation, *HF* heart failure, *HR* heart rate, *HT* hypertension, *Imp.* implanted, *IR-Injured* ischemia–reperfusion injured, *LA* large animal, *LAD-Flow* left anterior descending coronary flow, *LAP* left atrial pressure, *LV* left ventricular, *LVESP* left ventricular end-systolic pressure, *LVAD* left ventricular assist devices, *LVEDP* left ventricular end-diastolic pressure, *LVP* left ventricular pressure, *LVSP* left ventricular systolic pressure, *LVWT* left ventricular free wall thickness, *MAP* mean arterial pressure, *Max.* maximal, *MBP* mean blood pressure, *MEMS* microelectromechanical system, *Meth.* methodically, *mhealth* mobile health, *MI* myocardial infarction, *NGF-Inf.* nerve growth factor-infusion, *NICOM* non‐invasive cardiac output monitoring, *NP* nanoparticular, *PAP* pulmonary artery pressure, *PCWP* pulmonary capillary wedge pressure, *PDE-5* phosphodiesterase type 5, *PK/KD* pharmacokinetic/pharmacodynamic, *PV* pressure volume, *RDN* radiofrequency renal denervation, *ReDS* remote dielectric sensing, *Ref.* reference, *RPM* revolutions per min, *RV* right ventricular, *RV EDP* right ventricular end-diastolic pressure, *RVESP* right ventricular end-systolic pressure, *RVP* right ventricular pressure, *SA* small animal, *Saf.* safety, *TAU* time constant of relaxation, *TIC* Tachycardia induced cardiomyopathy, *V1/V2* vasopressine1/-2

### Selection of animal species and breeds

In 9 of 12 publications, the investigations were performed in conscious [15, 32, 33, 35–40, 43] and 3 in anesthetized animals [34, 37, 41, 42]. Most studies were performed in *canine* or *swine* models. Among the *canine* studies, 4 of 5 were conducted in *Beagle dogs* [32, 37, 40, 43]. In the remaining study, the race was not reported [39]. Among the *swine* models, 2 out of 5 studies were performed in *Yorkshire pigs* [15, 33], and 1 in *Landrace pigs* [38]. For the 2 remaining studies, the race was not reported [41, 42]. Two out of the remaining 3 studies were performed in *ovines*. Among these, the *Domestic sheep* [34] and the *Dorset sheep* were used [36]. In 1 publication, studies were conducted in *Cynomolgus monkeys* in addition to *Beagle dogs* [40].

### Telemetrically measurable parameters

Eleven of the devices used were implanted [15, 32–34, 36–41, 43] and 2 were applied to the body surface [32, 42]. In 1 publication, both wearable and implantable devices were used [32]. The most common parameters measured were: electrocardiogram (ECG) [15, 32, 33, 37–40], left ventricular pressure (LVP) [15, 32, 36, 37, 40, 43] and systemic blood pressure (BP) [15, 32, 37, 39, 40]. Further readouts were: HR [36, 41, 43], right ventricular pressure (RVP) [36], left atrial pressure (LAP) [34], cardiac output (CO) [32], contractility (+ dP/dt) and relaxation (− dP/dt) [43], anterior–posterior left ventricular chamber diameter (DAP) [37], left ventricular free wall thickness (LVWT) [37], left anterior descending- (LAD) and brachiocephalic (BC) flow [15] and pulmonary fluid concentration [42]. CO and pulmonary fluid concentration were determined by externally mounted devices while all other measured parameters were acquired by implanted sensors. The measurement of CO was performed using a noninvasive cardiac output monitoring technology (CHEETAH NICOM^™^, USA). This device was used in conscious animals, but since the data transmission is not telemetric it is only listed for the purpose of completeness [32].

### Study aims and disease models

**Fig. 3:**
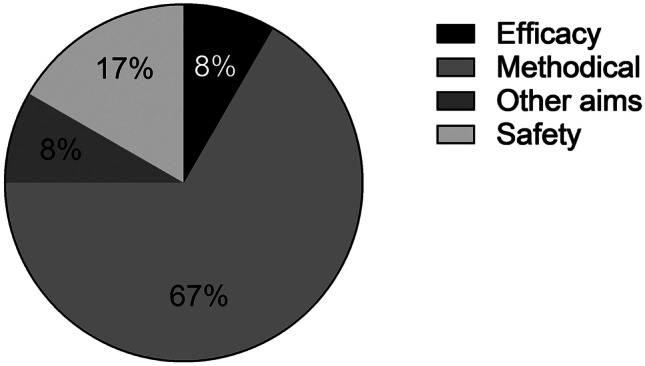
Study aims and their percentage distribution

Eight of 12 publications [15, 34, 36–38, 41–43] were classified as “methodical” (Fig. 3). Among these, 3 studies consisted of LA disease models enhanced with telemetry devices and tested for accuracy and robustness [15, 36, 38]. In 3 studies animal models were used to develop telemetry devices for continuous monitoring of cardiac functions in patients [34, 42, 43]. The remaining study investigated the feasibility and implantability of a left ventricular assist device (LVAD) with telemetric control and monitoring [41]. In 4 of the 8 “methodical” studies, the performance of the devices was validated by administration of already well-characterized substances such as phenylephrine [34], dobutamine/vasopressin [43], pimobendane [37], and furosemide [42]. Two of the 12 publications were classified with the term “safety”. In these, safety pharmacological investigations of various compounds on the CV function were conducted and changes in hemodynamics or cardiac function were recorded telemetrically [39, 40]. One publication evaluated the efficacy of the development candidate, pecavaptan (BAY1753011), in telemetered beagle dogs. It was therefore classified as “efficacy” [32]. The remaining study from publication 2 examined the effect of a protein on myocardial structural remodeling. It is therefore listed under “other aims”. Seven out of 12 studies included animal disease models. The most commonly used disease stimulus was pacing-induced HF [15, 32, 33, 36–38, 43]. In addition, HF was induced by volume overload due to atrioventricular- (AV-) fistula [15], pressure overload due to aortic banding [15], and cellular demise due to anterior myocardial infarction [42]. In the studies of the remaining 5 publications, healthy animals were used [32, 34, 39–41].

## Discussion

This review highlights the current status concerning the utilization of telemetry devices and their contribution to improving translational research for novel HF therapies. For this purpose, studies on preclinical HF research in telemetrically equipped LAs were included. Although the advantages and possibilities of such devices are obvious and also find active use in SA models [31, 44], only very few publications could be identified that apply these techniques in LA HF models (Fig. 2a). However, the potential and versatility of telemetry devices functioning as digital biomarkers in preclinical HF research are evident from these publications.

Various benefits by the use of telemetry devices in LA studies, but also for the use in patients, became clear. Basically, the measurement of hemodynamic and cardiac parameters becomes applicable in the conscious state. Established disease models, such as tachypacing-induced HF can be refined by these techniques and the confounder anesthesia can be eliminated. Considering the measured parameters, it becomes visible that, besides the standard parameters ECG and BP, a huge variety of parameters can be measured by means of telemetry devices. Thus, pressures in different areas of the heart, most commonly the LVP, as well as different vascular pressures can be determined simultaneously and continuously without the need for invasive surgery. The quality of the measurements is consistently rated as valid and robust among the publications. As telemetered animals are awake, they can move freely during experimental procedures like drug application and data collection. This further reduces animal stress and the associated activation of the adrenal medullary release of epinephrine and the sympathetic neuronal release of norepinephrine [45]. As a consequence, the use of telemetry devices results in key advantages compared to invasive measurements in anesthetized animals. This points out how non-invasive measurements can reduce confounders in data sampling.

The potential of telemetry measurements will be exemplified here by the conclusions of individual publications. Choy et al. (2014) were the first authors using a telemetry system (EndoGear1, Transonic Inc, Davis, CA, USA) to investigate the development and progression of HF pathogenesis induced by several common disease models (AV-fistula, aortic banding, tachypacing). LVESP and LVEDP as surrogates for HF, BP for systemic circulation and coronary flow for myocardial perfusion were measured telemetrically. The telemetry system was able to detect fluctuations and compensations that otherwise would have been missed during the entire investigational period by continuous recording. For instance, they could observe a temporary drop in BP as a feasible compensatory response during disease progression in aortic-banding models [15]. This points out the efficiency and reliability of such a system to register hemodynamic changes — thus functioning as a digital biomarker — without having any impact on physiological functions [15]. Regan et al. (2016) described a method to perform pressure volume (PV)-loop measurements, the gold standard for assessing cardiac function [46], in the conscious dog. For this purpose, they combined telemetry technique and sonomicrometry crystals which are integrated into a titanium skin button. This reduces the need for device maintenance as well as data drop-outs and allows the equipped animals to be used for an extended period of time [37]. Thus, detailed endpoints of cardiac function and hemodynamics can be investigated both, in single-dose studies and under prolonged exposure to the compound. In their experiment, Regan et al. were able to report both pathways of pimobendane, the increase in cardiac contractility and the peripheral arterial vasodilatation, demonstrating the validity of the measurements [37]. Their model can assess both, a decline in cardiac function as a basis to detect disease progression and an improvement in existing cardiac dysfunction [37]. The importance of such an approach is demonstrated by the myosin activator omecamtiv-mecarbil, which is currently further investigated in clinical trials [47, 48]. There is no effect on peripheral hemodynamics or LV contractility, but on parameters that are not routinely measured. Thus, LV systolic ejection time is increased without affecting myocardial oxygen consumption [49]. Amir et al.’s (2013) noninvasive remote dielectric sensing technology (Sensible Medical Innovations Ltd, Netanya, Israel) allows the quantification and detection of changes in lung fluid concentration, which can be used to indicate possible pulmonary congestion without the need to perform a computed tomography scan as well as an indication of the effect of diuresis. Benefits of new therapies on one of the cardinal symptoms of acute HF can thus be investigated or existing therapies can be optimized [42].

Although the validity of telemetry devices is evident, only one publication was found using such a technique to characterize a development candidate in a LA model. Mondritzki et al. (2020) compared their drug candidate pecavatan (BAY1753011), a dual V1/V2 receptor antagonist with the selective V2 receptor antagonist tolvaptan in a preclinical HF model in dogs. The telemetry system (Model L21, Data Sciences International, USA) recorded LVP and AP, as well as an ECG continuously in the conscious animal and provided accurate results throughout the entire study period. The authors concluded that extending the established tachypacing-induced canine HF model with a telemetry system delivers a more predictive model than invasive measurements in anesthetized animals performed in the same study [32]. Pecavaptan is currently being further investigated in the AVANTI Phase-II trial (A Multicenter, Randomized, Parallel Group, Double Blind, Active and Placebo Controlled Study of BAY1753011, a Dual V1a/V2 Vasopressin Receptor Antagonist, in Patients With Congestive Heart Failure) [50].

Despite these publications highlighting the usefulness and further potential of telemetry devices for translational research, a different development can be observed currently: Devices are being (further) developed for the use in patients and are therefore tested in animal models. This approach ranges from simple monitoring of end-stage HF patients to remote monitoring and control of cardiac functions. The development is powered by the success of the CardioMEMES^™^ device, a microelectromechanical system for remote patient monitoring. The CHAMPION trial (CardioMEMS Heart Sensor Allows Monitoring of Pressure to Improve Outcomes in New York Heart Association Class III Heart Failure Patients) impressively demonstrated that a medical device, additionally to conventional pharmacotherapy, is able to achieve a significant reduction in hard clinical endpoints such as hospitalization rate [51]. Perl et al. (2019) were able to show that the V-LAP device (Vectorious medical technologies, Tel Aviv, Israel) provides accurate and robust monitoring of congestion in the pulmonary circulation by direct pressure measurement in the left atrium. Telemetric measurements showed an excellent correlation with invasively measured pulmonary capillary wedge pressure (*R* = 0.95). This method may allow a more accurate assessment of pulmonary congestion than pressure measurements in the pulmonary arteria as performed with the FDA-approved CardioMEMS^™^ device. Mondritzki et al. (2018) were able to use the LV-MEMS device (St. Jude Medical, Saint Paul, USA) to assess LV performance in a canine HF model by means of microelectromechanical sensors [35]. They were able to prove the validity of their method by reliably registering the pharmacological effects of dobutamine and vasopressin. Direct pressure measurement in the LV is accompanied by potential advantages over measurements in the right heart or pulmonary arteria. As LV pressure rises earlier than pulmonary arterial pressure in acute HF which may also be influenced by other factors, such as pulmonary disease, an earlier and more specific diagnosis of cardiac decompensation is possible with LV-MEMS [35]. Siavash S Asgari et al. (2013) developed the UMC-Physio Device, a wirelessly chargeable LVAD with telemetric HR monitoring and adjustment of the device’s revolutions per time (RPM). The physician is also able to monitor and control the LVAD via an internet connection [41].

The importance of such devices for clinical practice lies in the monitoring of cardiac and hemodynamic functions in an ambulatory setting [34]. Thus, highly personalized HF management based on objectively collected data becomes possible. This may also include daily adjustment of medication such as diuretic requirements. Drug-related problems can thus be reduced and consequently drug therapy safety increased. In addition, acute events and exacerbations can be detected early, hospitalizations can be reduced, and the patient’s quality of life can be improved [34, 52–54].

A gap is forming in the implementation of digital biomarkers in drug development. While the usage of telemetry devices in SA pharmacological models is meanwhile proving valuable [55], their usage in LAs still seems to be restrained. In the clinical sector, on the other hand, precisely such mhealth technologies are most prominently discussed for future medical development and are continuing to rise [18]. Thus, demonstrating their potential to evaluate the effects of drugs more evidently than with conventional methods, it is only obvious that clinical trials of new drugs will also be increasingly supported by mhealth devices in the future. Consequently, they must also be applied in the preceding preclinical phase of drug discovery.

## Conclusions

In summary, commercially available telemetry devices enable the reliable and robust recording of cardiac and hemodynamic functions in animal (disease) models. In addition, there are already promising approaches implementing such technologies in patients. The ability to collect data, due to the utilization of telemetry devices, continuously while the subject is awake provides a more predictive approach to evaluate drug effects and side effects than traditional invasive measurement techniques. The incorporation of the mentioned mhealth devices in both, preclinical and clinical testing of novel compounds, represents a translational approach that offers the opportunity to increase the validity of animal models and further reduces failures in the cost-intensive late-stage development of drugs. This review also represents a recommendation to advance such approaches in the development of new, effective HF therapies to manage the increasing medical demand.
